# Supplementary material for: In-situ observation of silk nanofibril assembly via graphene plasmonic infrared sensor
Source: Nat Commun. 2024 May 31;15:4643. doi: 10.1038/s41467-024-49076-5 (PMC11143229; doi:10.1038/s41467-024-49076-5)
Supplement: Supplementary file 1 — Supplementary Information [file 41467_2024_49076_MOESM1_ESM.pdf]

Supplementary Information for

"In-situ Observation of Silk Nanofibril Assembly via Graphene plasmonic infrared sensor"

Chenchen Wu[1, 2, 3], *Yu Duan*[1, 2, 4], *Lintao Yu*[5, 6], *Yao Hu*[7], *Chenxi Zhao*[5, 6], *Chunwang Ji*[1, 2], *Xiangdong Guo*[1, 2, 3, 8], *Shu Zhang*[1, 2, 3], *Xiaokang Dai*[1, 2, 3], *Puyi Ma*[1, 2, 3], *Qian Wang*[7]*, *Shengjie Ling*[5, 6]*, *Xiaoxia Yang*[1, 2, 3]*, *and Qing Dai*[1, 2, 3, 8]*

[1]CAS Key Laboratory of Nanophotonic Materials and Devices, National Center for Nanoscience and Technology, Beijing 100190, China

[2]CAS Key Laboratory of Standardization and Measurement for Nanotechnology, National Center for Nanoscience and Technology, Beijing 100190, China

[3]Center of Materials Science and Optoelectronics Engineering, University of Chinese Academy of Sciences, Beijing 100049, China

[4]Henan Institute of Advanced Technology, Zhengzhou University, Zhengzhou 450001, China

[5]School of Physical Science and Technology, ShanghaiTech University, Shanghai 201210, China

[6]Shanghai Clinical Research and Trial Center, Shanghai 201210, China

[7]Department of Physics, University of Science and Technology of China, Hefei, Anhui 230026, China

[8]School of Materials Science and Engineering, Shanghai Jiao Tong University, Shanghai, 200240, China

These authors contributed equally: Chenchen Wu, Yu Duan.

E-mail: daiq@nanoctr.cn, yangxx@nanoctr.cn, lingshj@shanghaitech.edu.cn, wqq@ustc.edu.cn

This PDF file includes:

Supplementary Notes 1-5

Supplementary Figures S1-S19

Supplementary References

Supplementary Note 1: The dispersion of graphene plasmon is[1, 2]:

$$\omega_{pl}=\sqrt{\frac{e^2|E_F|q}{2\pi\hbar^2\varepsilon_0\varepsilon_r}},$$

where ε_0 is the dielectric constant of air, ε_r is the average dielectric constant of its surrounding medium, E_F is the fermi energy of graphene, q is the wave vector and $q=\pi/W$ (W is the width of graphene nanoribbon). Thus, the resonance frequency of graphene plasmon (ω_{pl}) is related to W and E_F of graphene nanoribbon. In addition, when the width of graphene nanoribbon is close to the spacing between them, the energy loss due to plasmon-plasmon interactions between adjacent ribbons is minimized. This optimizes the extinction of graphene plasmons, a crucial factor for our sensor's performance[3]. To achieve the highest sensitivity for SNF assembly detection, we carefully designed the W and E_F (proportional to ΔV_G, $\Delta V_G=|V_G - V_{\mathrm{CNP}}|$) in a cooperative manner. A larger W requires a higher ΔV_G to reach the desired resonance frequency for optimal overlap with the fingerprint region of target molecules[4]. However, applying a high ΔV_G carries the risk of device breakdown, potentially compromising subsequent measurements[5]. Therefore, we strategically chose W of approximately 60 nm and period-to-width ratio of 2:1 to balance these considerations.

As shown in Figure S5b, d, and f, the initial doping level of graphene can vary between sensors due to fabrication processes, leading to different charge neutral points (V_{CNP}). To address this, we performed I_{SD}-V_G characterization to determine the V_{CNP}, and measured the background FTIR transmittance (T_0) at V_{CNP}. We then get Extinction spectrum (Extinction=1-T_{V_G}/T_0, where T_{V_G} represents the transmittance measured at a specific gate voltage (V_G)) by tuning the gate voltage (V_G) to achieve the desired resonance frequency that overlaps with the Amide I band. Figure S5i exemplifies this process, showing the dynamic response of graphene plasmon from 1450-1750 cm^{-1} as ΔV_G is swept from 59 V to 119 V. Ultimately, we select the appropriate gate voltage (ΔV_G =89 V, blue curve) for subsequent FTIR measurements and analysis, where the graphene plasmon response best aligns with the target molecule's fingerprint region.

Supplementary Note 2: Extracting length of SNFs by deep learning algorithm

We use Python to convert the picture into a binary graph using the formula S1. The Python code has been attached in Source Data. Then Hough line detection is applied to calculate the line segment direction in the graph, and the actual length is obtained by calculating the pixel points along the line segment direction:[6]

$$\mathrm{Dst}(x,y)=\begin{cases} \text{mavel} & \text{if } \mathrm{src}(x,y) > \text{thresh} \\ 0 & \text{otherwise} \end{cases} \quad \text{(S1)}$$

Supplementary Note 3: Analyzing secondary structure content of SNFs.

To analyze the secondary structure content of SNFs, we acquire the average ΔExtinction spectrum (e.g., Fig. S8). Notably, we utilize 3-4 Extinction spectra derived from multiple FTIR measurements. ΔExtinction spectra are obtained by subtracting individual Extinction spectra of SNFs with graphene plasmon enhancement from their respective baselines. Each baseline is established through asymmetric least squares fitting in reference to the Extinction spectra. Subsequently, we compute the average ΔExtinction spectrum using these individual ΔExtinction spectra. The average ΔExtinction spectrum is fitted using second-derivative peak-finding and Gaussian line patterns, where the peak in the range of 1616-1637 cm^{-1} belongs to β-sheets, 1638-1662 cm^{-1} belongs to Random coils, and 1663-1685 cm^{-1} belongs to turns[7]. The different secondary structure contents are obtained by analyzing the peak's area ratio. It is noticed that the analysis of the spectra with graphene plasmon enhancement (red curve) is consistent well with the spectra without graphene plasmon enhancement (blue curve), as depicted in Fig. S14.

Supplementary Note 4: SNF assembly with and without graphene.

SNF assembly with graphene: The graphene plasmonic infrared sensor and SF solution are preheated for 10 minutes at the set temperature respectively. Then the sensor is placed in the SF solution with the graphene surface facing down to avoid the deposition of SF and impurities on the graphene surface due to gravity. After assembling, the sensor is cleaned with deionized water, and then the surface is blown dry with a nitrogen gun.

SNF assembly without graphene: The SF solution is diluted with deionized water to a concentration of 0.1% weight, gently agitated to ensure uniformity, and then incubated at 333 K in an oven for a period ranging from 7 days to 1 month. The SNFs are fully assembled with a birefringent effect.

Supplementary Note 5: All-atom molecular dynamics (MD) simulations

We utilize the following peptide sequence as a fragment of SF in the all-atom MD simulations:

GAGAGSGAGAGSGAGAGSGAGAGSGAGAGSGAGAGYGAGVGVGYGAGYGAGAGAGYG
AGAGSGAASGAGAGSGAGAGSGAGAGSGAGAGSGAGAGSGAGAGSGAGAGSGAGAGS

GAGAGSGAGAGSGAGVGSGAGAGSGAGAGVGYGAGAGVGYGAGAGSGAASGAGAGSG
AGAGSGAGAGSGAGAGSGAGAGSGAGAGSGAGAGSGAGAGSG

Periodic boundary conditions (PBC) are implemented for all models. Firstly, the steepest descent method is applied to perform energy minimization, in which the step-size is 0.01 nm and the convergence value of the maximum force is 100 $kJ\cdot mol^{-1}\cdot nm^{-1}$. Then 1 ns pre-equilibrium simulations are performed to relax each system. Finally, production simulations are performed for each system, the time scale is as long as 1 μs. For MD simulations, the time steps are set to 2 fs; leap-frog algorithm[8] is applied to integrate the equation of motion; SETTLE algorithm[9] is applied to constrain water molecules as rigid; LINCS algorithm[10] is applied to constrain the length of covalent bonds connected to H atoms. PME algorithm[11] is applied to calculate Coulomb interaction, the real space cut-off is set to 1.0 nm. The Lennard-Jones (L-J) cut-off is set to 1.0 nm, and the long-range dispersion corrections for energy and pressure are applied. Both the pre-equilibrium simulations and the production simulations are performed under the NPT ensemble. Velocity-rescale algorithm[12] is applied to control the temperature of the protein and the non-protein part separately, the time constant is set to 0.2 ps. Berendsen algorithm[13] is applied to control the pressure for pre-equilibrium simulations, the time constant is set to 0.5 ps, the reference pressure is set to 1.01325 bar, and the compressibility coefficient is set to 4.5×10^{-5} bar^{-1}. Parrinello-rahman algorithm[14] is applied to control the pressure for production simulations, the time constant is set to 2.0 ps; other parameter details are the same as those of the pre-equilibrium simulation. It is important to highlight that, for graphene-containing systems, the pressure coupling algorithm is executed in a semi-isotropic manner. The initial SF configurations are derived from those obtained in the preceding production simulations.

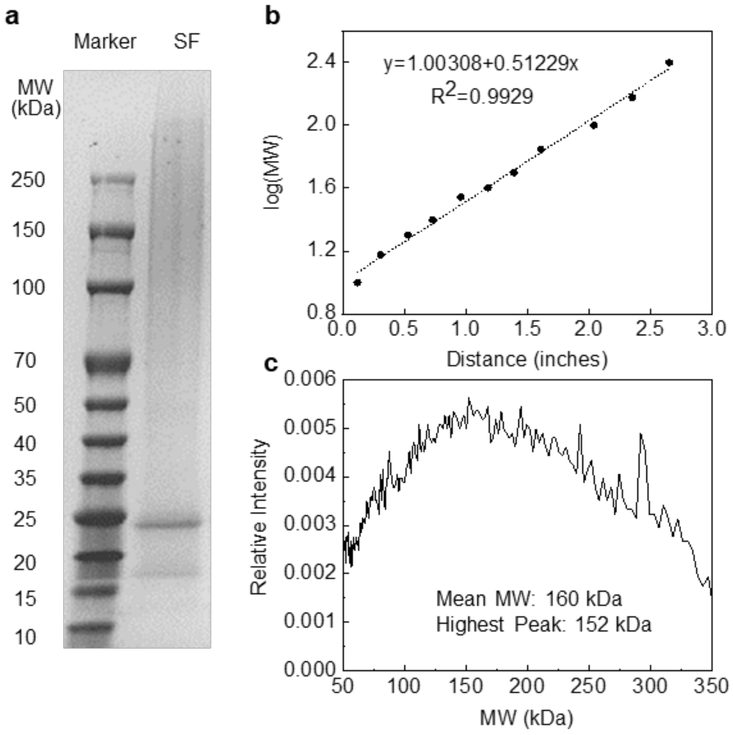

Supplementary Figure S1 Characterization the molecular weight (MW) of silk fibroin (SF). (a) Polyacrylamide gel images of SF with reference protein ladder. The SF solution, with a concentration of 1.65% (*w/w*, weight in weight), was blended with loading buffer and subjected to electrophoresis at 120 V for 80 minutes. (b) The relation between the log (MW) and the distance determined through simple linear regression analysis (R^2 =0.9929). This analysis yielded a linear equation, which was then applied to convert the distances measured in inches on the gel images to MW values for the sample lanes. (c) The MW distributions of SF with highest intensity peak at 152 kDa and mean MW of ~160 kDa. This distribution is broad due to the SF molecules in the spinning stock solution forming a fractal network structure[15].

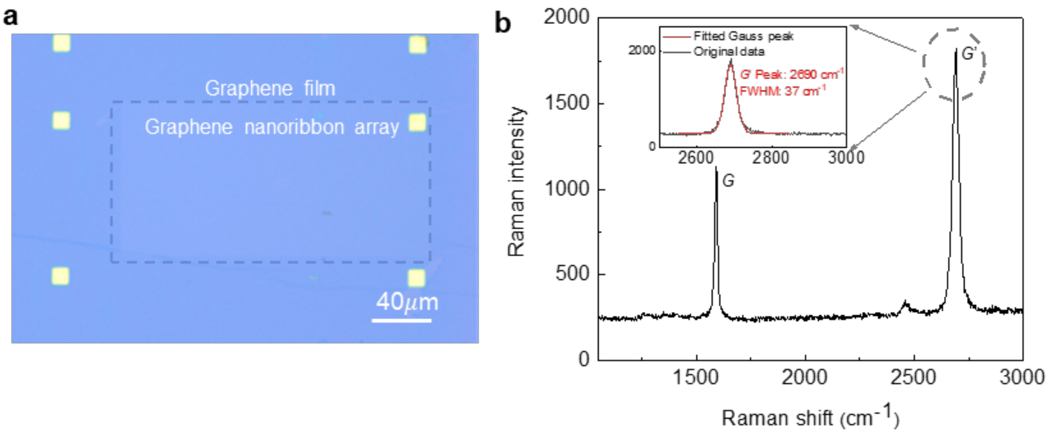

Supplementary Figure S2 Characterization of graphene plasmonic infrared sensor. (a) Optical microscope image of graphene film and graphene nanoribbon region, which shows the graphene is continuous and uniform. (b) Raman spectrum of utilized graphene film. 514 nm laser is used. By fitting the Raman spectrum of graphene we measured, as magnified in the left box, the *G*' peak is observed to be a single peak, confirming that it is single layer graphene[16-18].

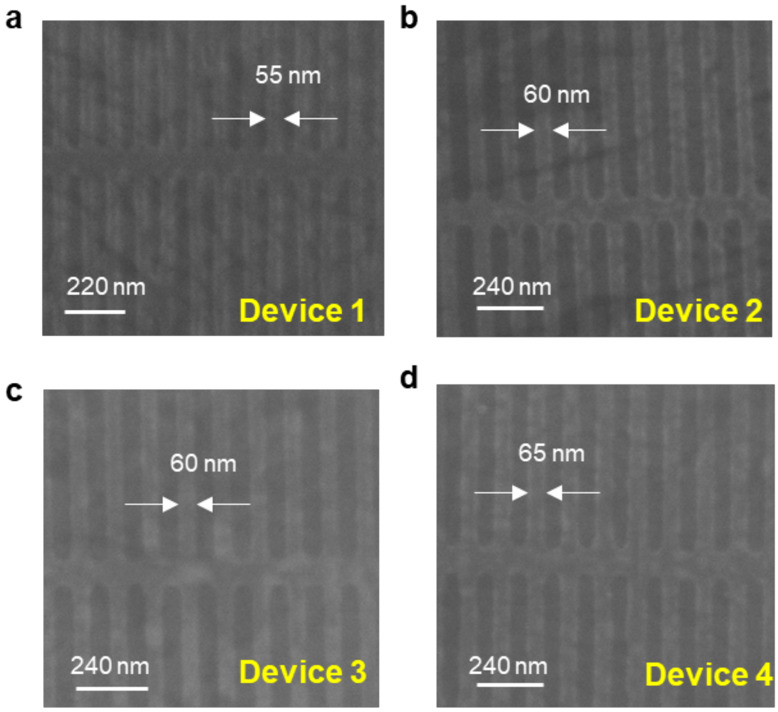

Supplementary Figure S3. Scanning electron microscope images of graphene nanoribbons. (a) Device 1 as utilized in Figure 1c. The graphene nanoribbon width is 55 nm, and period is 110 nm. (b) Device 2 as utilized in Figure 2a&3b. The graphene nanoribbon width is 60 nm, and period is 120 nm. (c) Device 3 as utilized in Figure 2b&3c. The graphene nanoribbon width is 60 nm, and period is 120 nm. (d) Device 4 as utilized in Figure 2c&3d. The graphene nanoribbon width is 65 nm, and period is 120 nm.

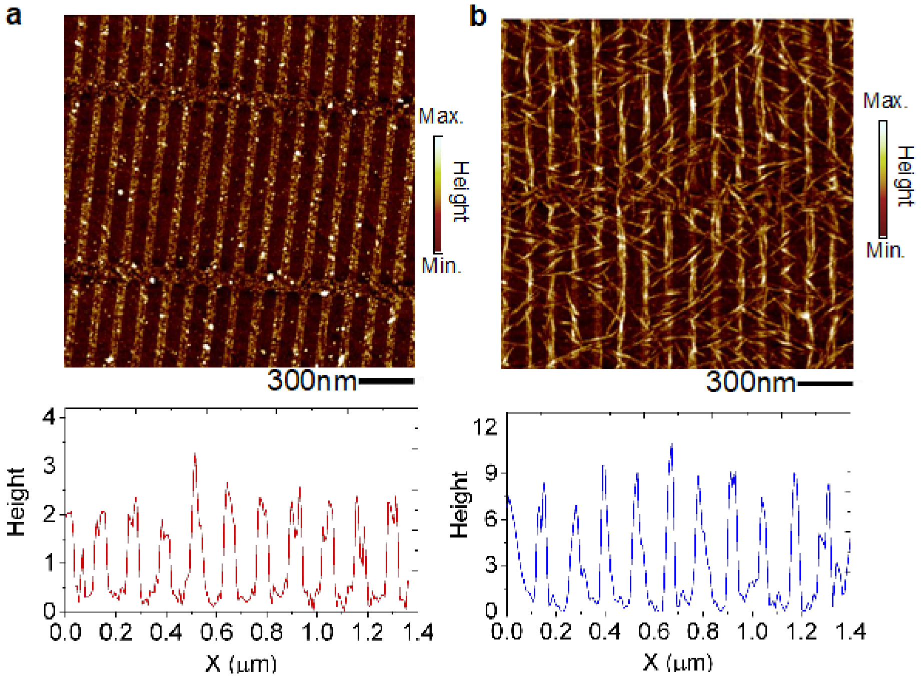

Supplementary Figure S4. The AFM data of graphene nanoribbons with and without SNFs. (a) The morphology and extracted height of bare graphene nanoribbons. Although the theoretical thickness of a monolayer of graphene is 0.34 nm, our AFM measurements indicate that the height of the monolayer graphene exceeds 1 nm. This discrepancy is attributed to the use of poly(methyl methacrylate) (PMMA) during the fabrication of graphene plasmonic devices[19], which results in 1-2 nm thick PMMA residues on the graphene surface. These residues are challenging to completely eliminate[20, 21]. (b) The morphology and extracted height of graphene nanoribbons with SNFs.

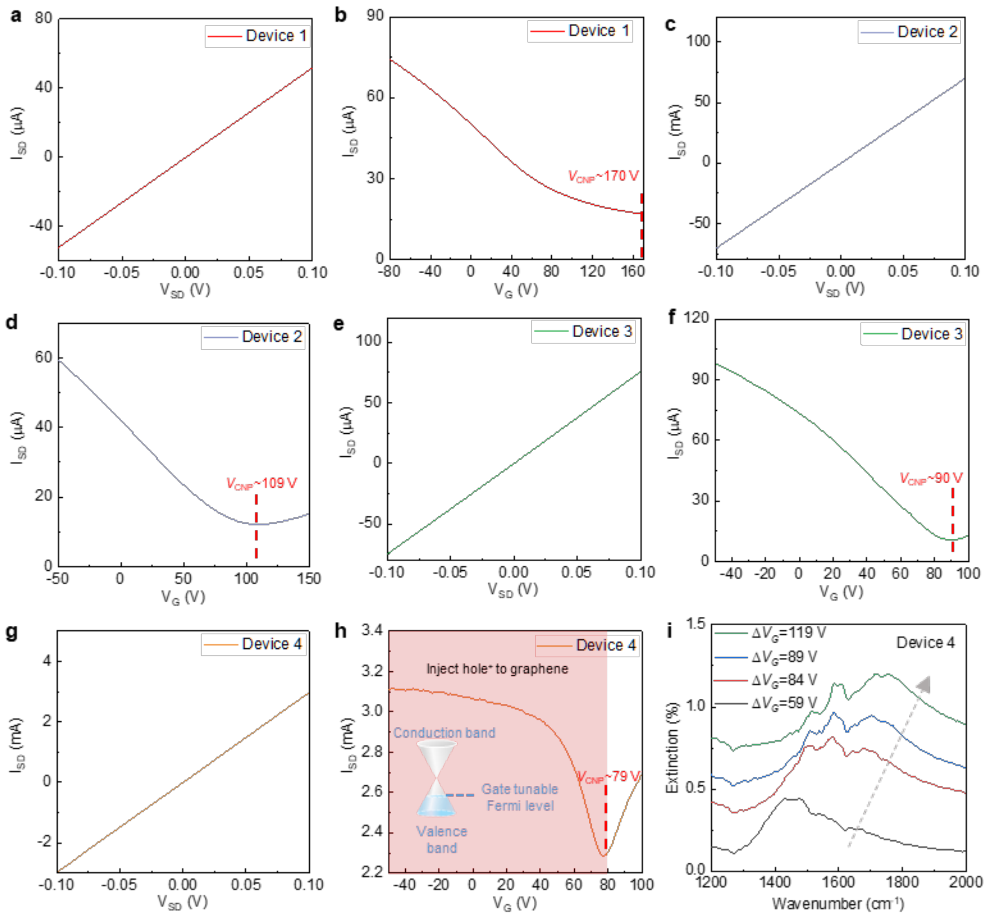

Supplementary Figure S5. Electrical performance of graphene plasmonic infrared sensors. (a) The I_{SD}-V_{SD} data, and (b) I_{SD}-V_G data of the graphene plasmonic sensor (Device 1), as utilized in Fig. 1c. (c) The I_{SD}-V_{SD} data, and (d) I_{SD}-V_G data of the graphene plasmonic sensor (Device 2), as utilized in Fig. 2a&Fig. 3b. (e) The I_{SD}-V_{SD} data, and (f) I_{SD}-V_G data of the graphene plasmonic sensor (Device 3), as utilized in Fig. 2b&Fig. 3c. (g) The I_{SD}-V_{SD} data, and (h) I_{SD}-V_G data of the graphene plasmonic sensor (Device 4), as utilized in Fig. 2c&Fig. 3d. (i) The tunable graphene plasmon when varying gate voltage (V_G) relative to charge neutrality point (V_{CNP}) ($\Delta V_G = V_G - V_{\mathrm{CNP}}$) measured by Device 4. V_{SD} and I_{SD} denote the applied voltage and the measured current between the source and drain, respectively. V_{SD}-I_{SD} curves were recorded with V_G set to 0 V, while V_G-I_{SD} curves were obtained with V_{SD} at 0.1 V. The linear slope observed in the V_{SD}-I_{SD} curve suggests good contact between the graphene and the electrodes. The lowest point on the V_G-I_{SD} curve indicates the

V_{CNP}. As shown in Fig. S4h, applying a V_G lower than the V_{CNP} (indicated by the red shaded area) shifts the Fermi level towards the valence band, indicating p-doping of graphene.

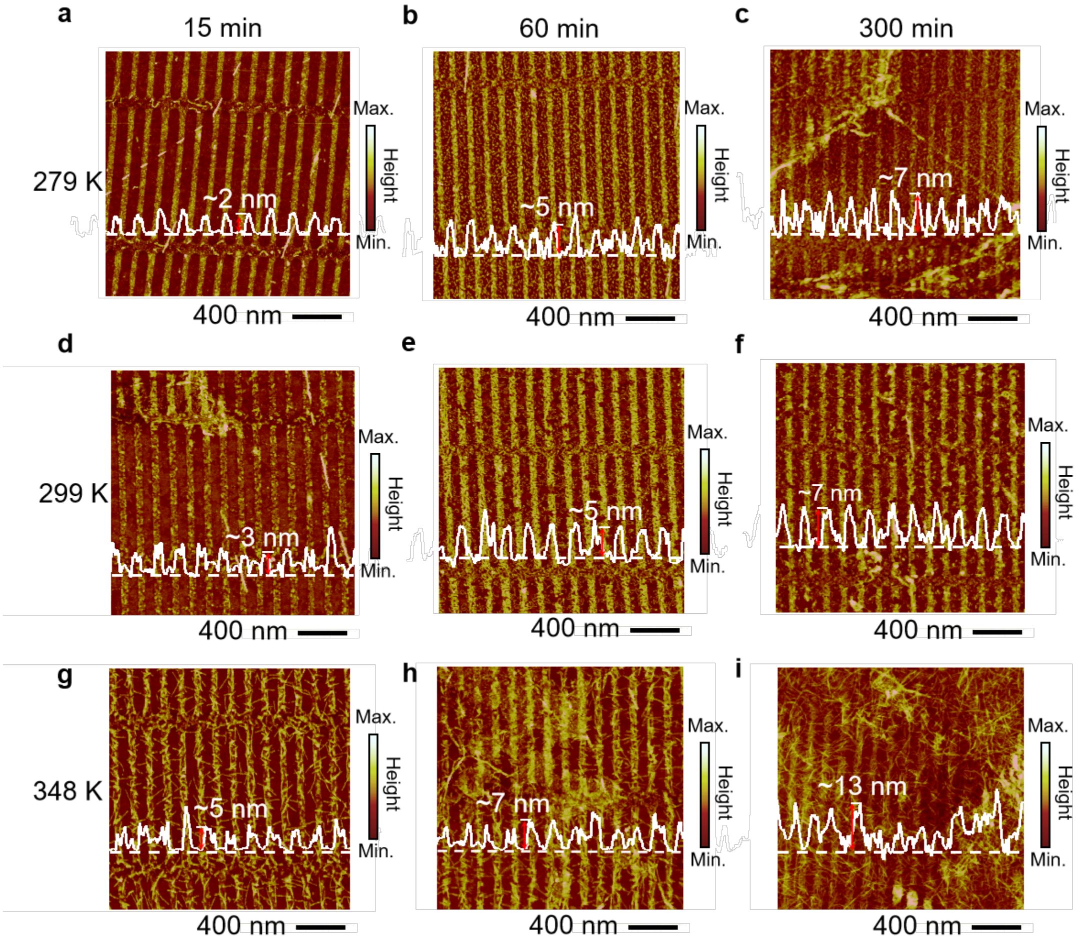

Supplementary Figure S6. The AFM data of intermediates and SNFs on graphene nanoribbons at different temperatures. At 279 K with an assembly duration of (a) 15 minutes, (b) 60 minutes, and (c) 300 minutes. At 299 K with an assembly duration of (d) 15 minutes, (e) 60 minutes, and (f) 300 minutes. At 348 K with an assembly duration of (g) 15 minutes, (h) 60 minutes, and (i) 300 minutes.

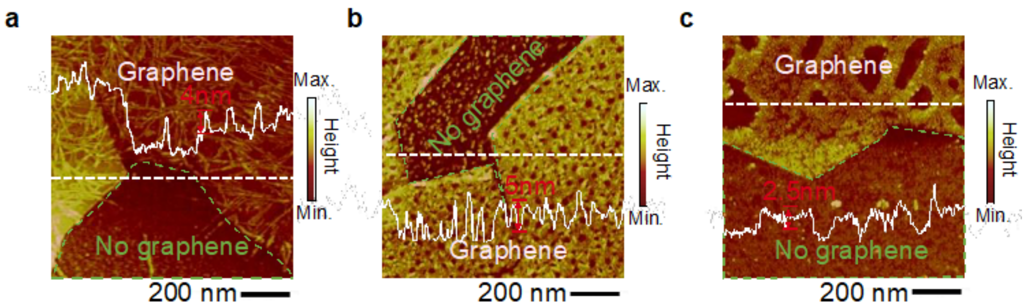

Supplementary Figure S7. The AFM data of assembled SF on mechanically exfoliated graphene at different temperatures. (a)348 K, (b)299 K, and (c)279 K. The area highlighted with a green dashed line is the SiO_2 substrate, and the rest is the SNFs on graphene. The concentration of SF solution is 10.1 µg/mL, and the assembly duration is 12 hours. The white dashed line highlight the sampling position of height.

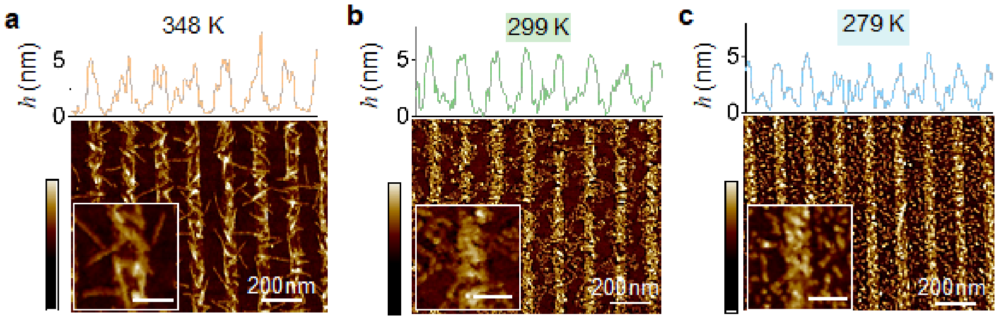

Supplementary Figure S8. The assembled SNFs on graphene at different temperatures. AFM data of 3-4 nm thick SNFs on graphene nanoribbons at (a) 348 K. (b) 299 K, and (c) 279 K. The scalebar is 200 nm.

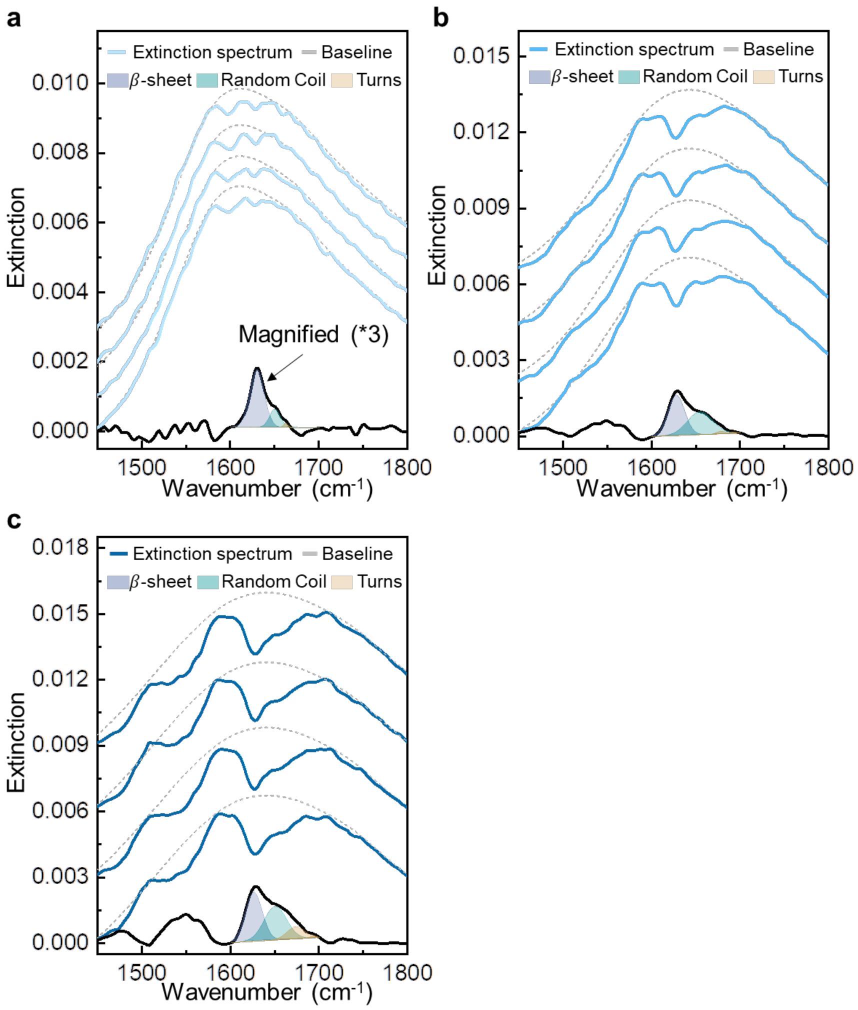

Supplementary Figure S9. Graphene plasmon-enhanced FTIR with different assembly durations at 279 K. The assembly duration is (a) 15 minutes. (b), 60 minutes, and (c) 300 minutes. In each figure, the colored curves are the original extinction spectra of graphene plasmon with assembled SNFs, which is repetitively measured at same ΔV_G. The dashed grey curves are the baselines. The black curve is the average ΔExtinction spectra for secondary structure analysis. The spectrum were measured at ΔV_G=130 V(a), 230 V(b), 210 V(c), respectively. $\Delta V_G = V_G - V_{CNP}$.

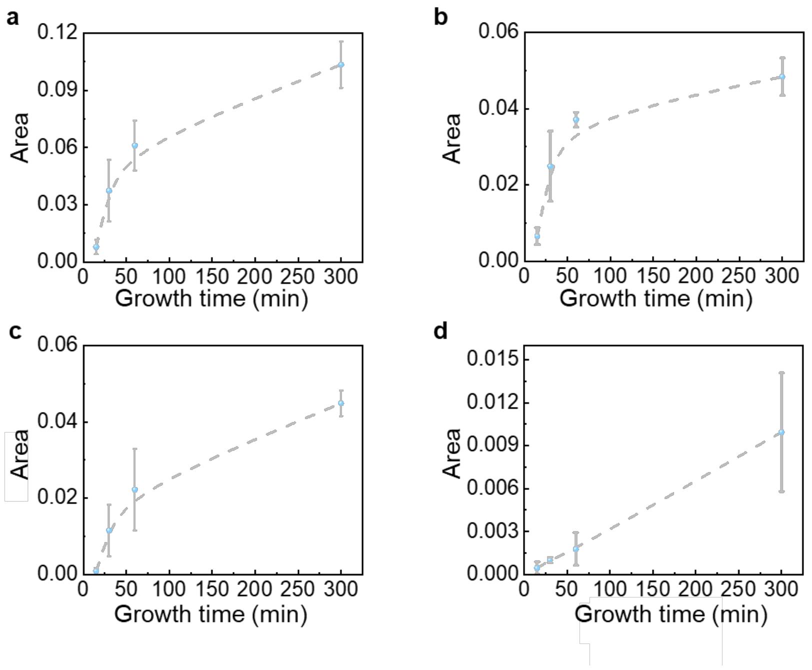

Supplementary Figure S10. The extracted secondary structure area with different assembly durations at 279 K. The average graphene plasmon-enhanced area with standard error of (a) amide I band, (b) β-sheet, (c) random coil, and (d) turn extracted from Figure S9. The grey dashed lines are the guideline. The data were collected from four individual measurements of graphene plasmon-enhanced FTIR and expressed as as mean values +/- SEM.

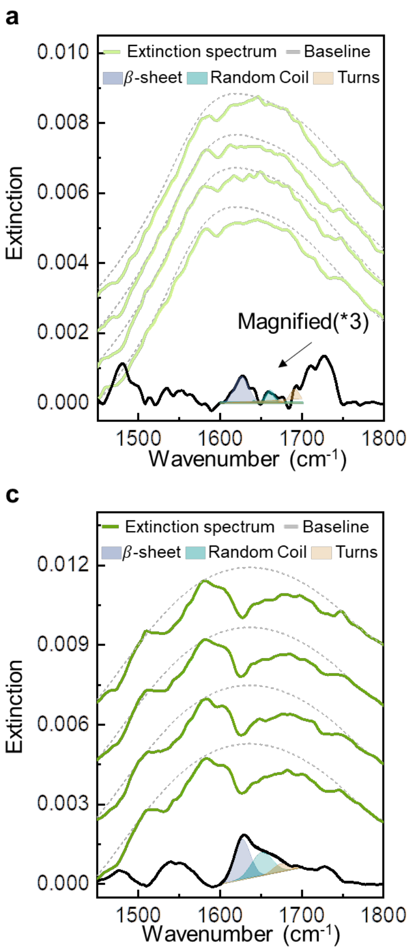

Supplementary Figure S11. Graphene plasmon-enhanced FTIR with different assembly durations at 299 K. The assembly duration is (a) 15 minutes, (b) 60 minutes, and (c) 300 minutes. The spectrum were measured at ΔV_G=150 V(a), 270 V(b), 250 V(c), respectively. In each figure, the colored curves are the original extinction spectra of graphene plasmon with assembled SNFs, which is repetitively measured at same ΔV_G.

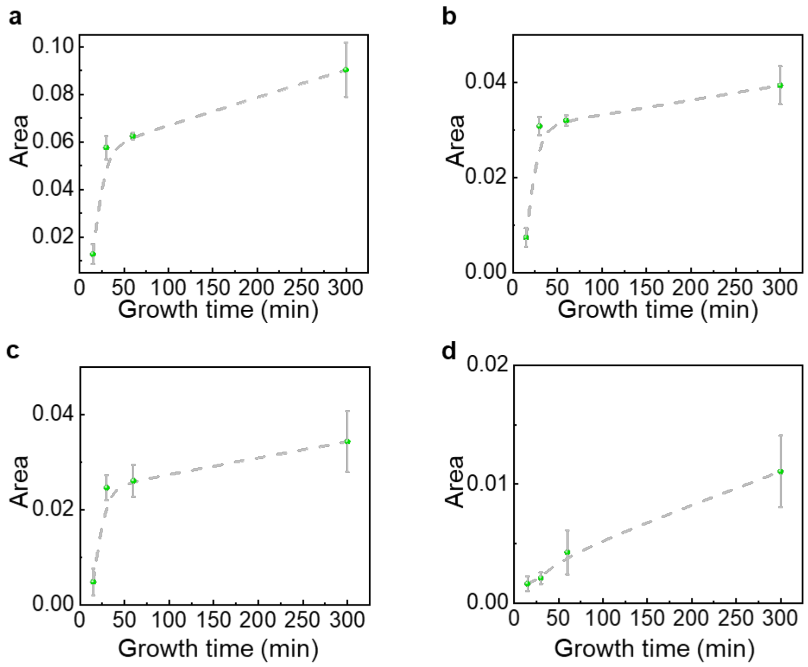

Supplementary Figure S12. The extracted secondary structure area with different assembly durations at 299 K. The average graphene plasmon-enhanced area with standard error of (a) amide I band, (b) β-sheet, (c) random coil, and (d) turn extracted from Figure S7. The grey dashed lines are the guideline. The data were collected from four individual measurements of graphene plasmon-enhanced FTIR and expressed as as mean values +/- SEM.

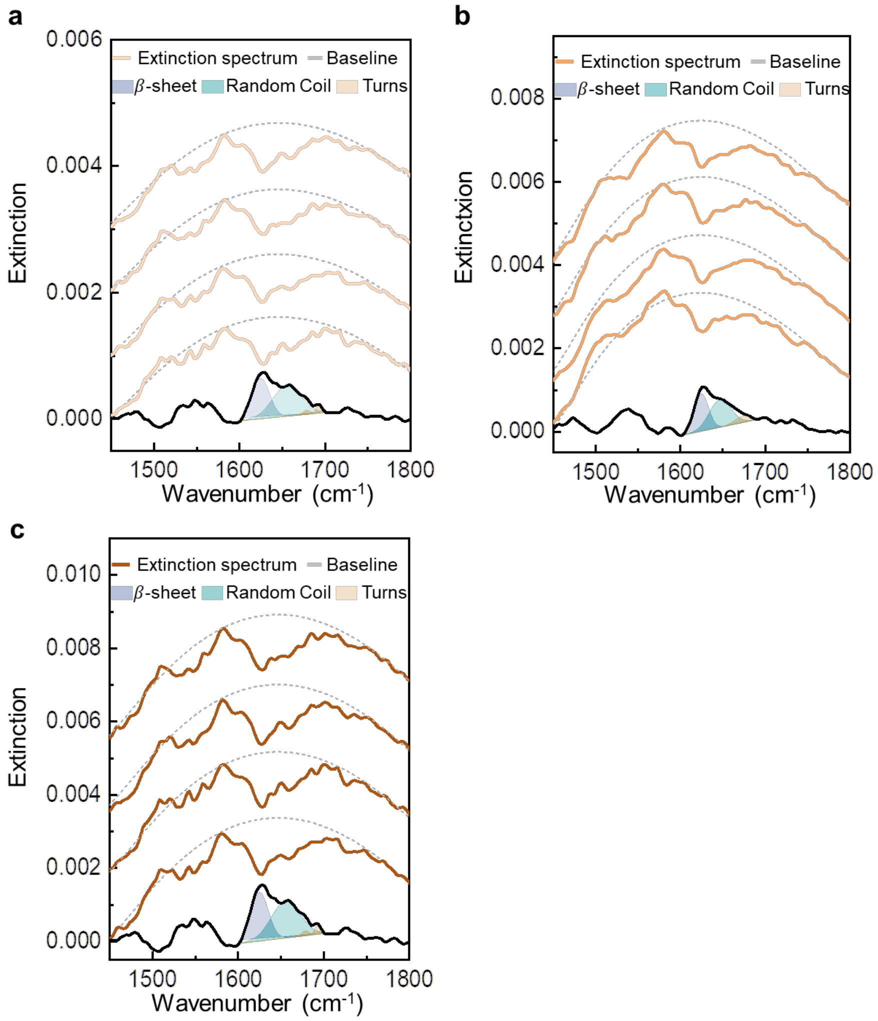

Supplementary Figure S13. Graphene plasmon-enhanced FTIR with different assembly durations at 348 K. The assembly duration is (a) 15 minutes, (b) 60 minutes, (c) 300 minutes. The spectrum were measured at ΔV_G=9 V(a), 104 V(b), 84 V(c), respectively. In each figure, the colored curves are the original extinction spectra of graphene plasmon with assembled SNFs, which is repetitively measured at same ΔV_G.

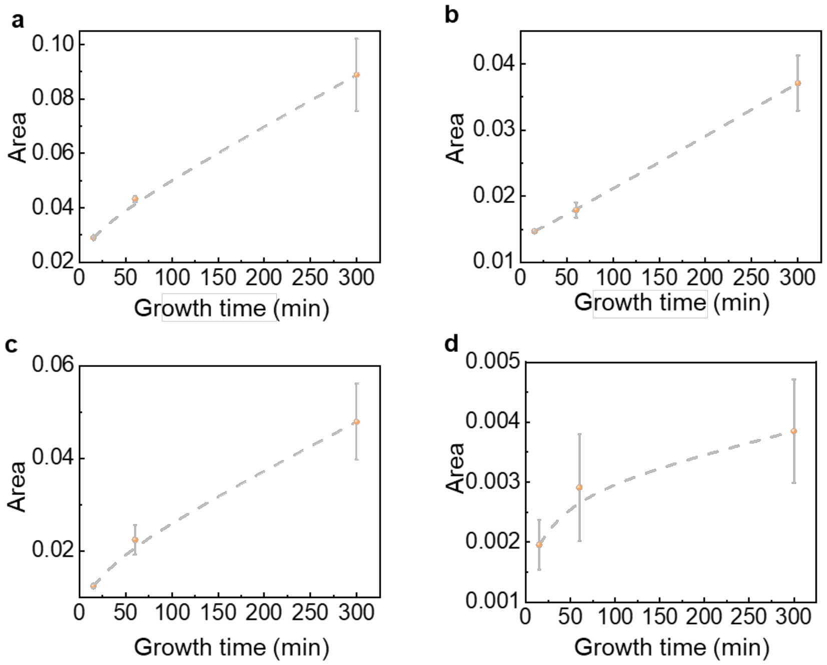

Supplementary Figure S14. The extracted secondary structure area with different assembly durations at 348 K. The average graphene plasmon-enhanced area with standard error of (a) amide I band, (b) β-sheet, (c) random coil, and (d) turn extracted from Figure S5. The grey dashed lines are the guideline. The data were collected from four individual measurements of graphene plasmon-enhanced FTIR and expressed as as mean values +/- SEM.

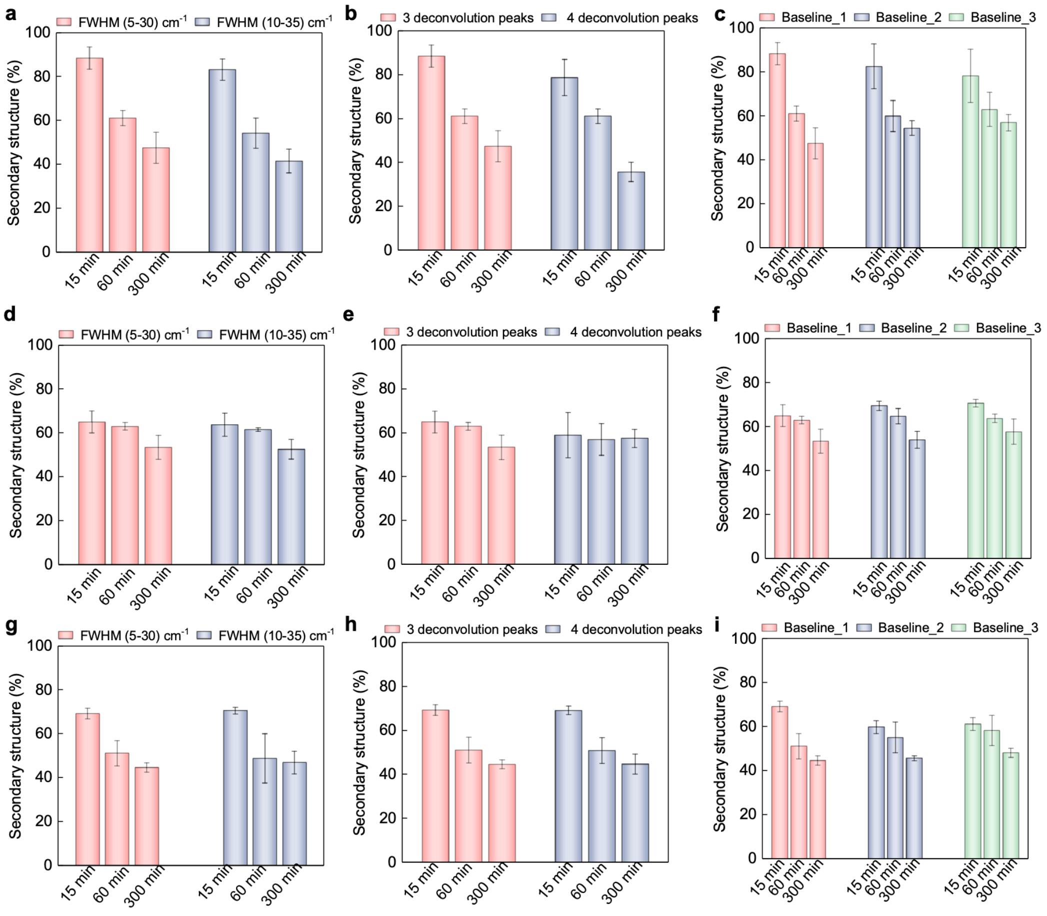

Supplementary Figure S15. The change of β-sheet content at different assembly durations using different parameters. (a)-(c) 279 K. (d)-(f) 299 K. (g)-(i) 348 K. It is noted that the secondary structure content is varied because different fitting parameters are utilized, such as different baselines, number and full width at half maximum (FWHM) of deconvolution peaks. By summarizing the fitting results with average value with standard error, we observed that the β-sheet content consistently decreases with increased assembly durations. The data were collected from four individual measurements of graphene plasmon-enhanced FTIR and expressed as as mean values +/- SEM.

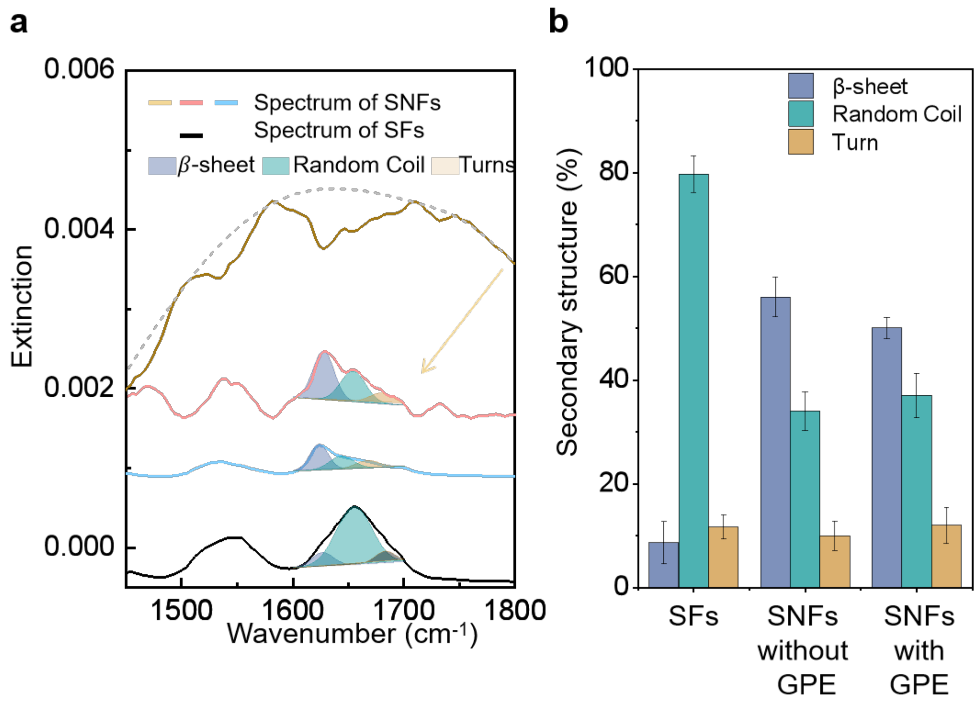

Supplementary Figure S16. Secondary structure content analysis of SFs and SNFs. (a) Experimental extinction spectra include the extinction spectrum of ~7 nm-thick SNFs assembled on graphene with graphene plasmon enhancement (GPE) (yellow curve), the extracted ΔExtinction spectrum (red curve), the extinction spectrum of ~7 nm-thick SNFs assembled on graphene without GPE (blue curve), and extinction spectrum of SFs (black curve). The grey dashed curve is the baseline. (b) Extracted average content with standard error of different secondary structures from (a). The data were collected from four individual measurements and expressed as as mean values +/- SEM.

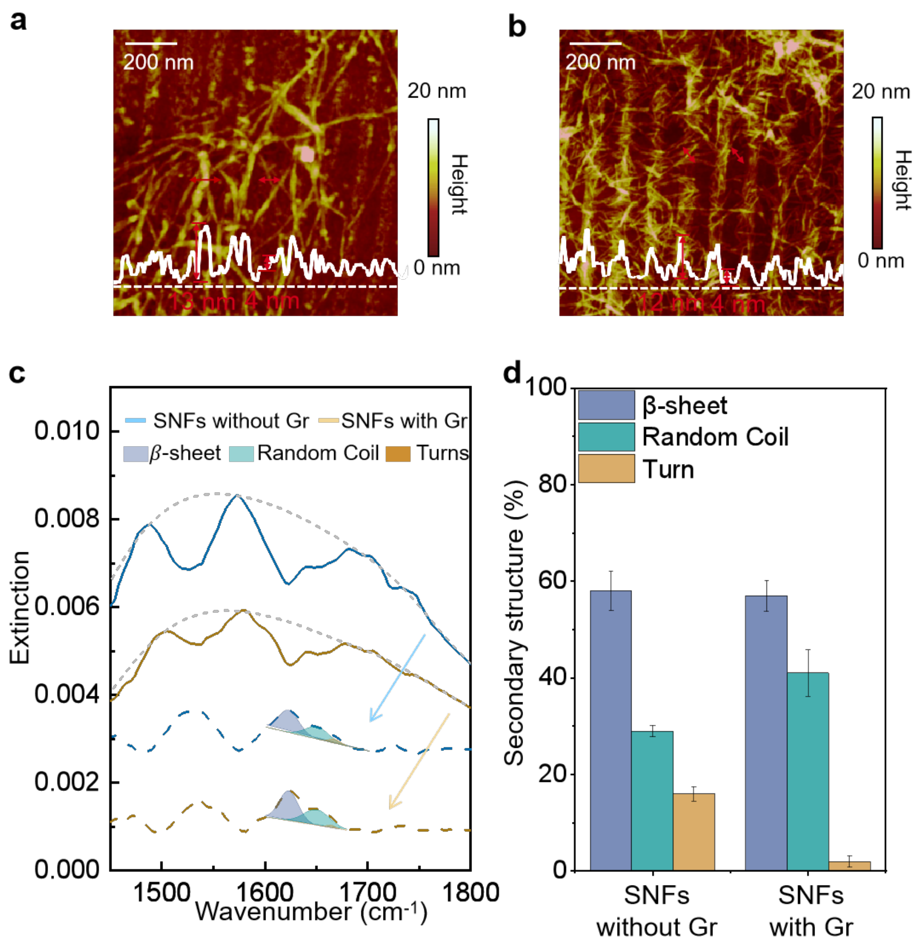

Supplementary Figure S17. Secondary structure contents of SNFs obtained with different preparation methods. (a) The morphology of assembled SNFs without graphene. The thickness of a single SNF falls within the range of 4-13 nm. (b) The morphology of assembled SNFs with graphene. SNFs were prepared by immersing a graphene plasmonic infrared sensor in SF solution for 300 minutes to facilitate assembly (details in Note 4). The thickness of the resulting SNFs also lies within the range of 4-12 nm. (c) Extinction spectra of SNFs without graphene (blue curve) and with graphene (yellow curve) and ΔExtinction spectra (blue dashed curve, yellow dashed curve). The grey dashed curve denotes the baseline. (d) The average content with standard error of different secondary structures extracted from (c). The data were collected from four individual measurements and expressed as as mean values +/- SEM.

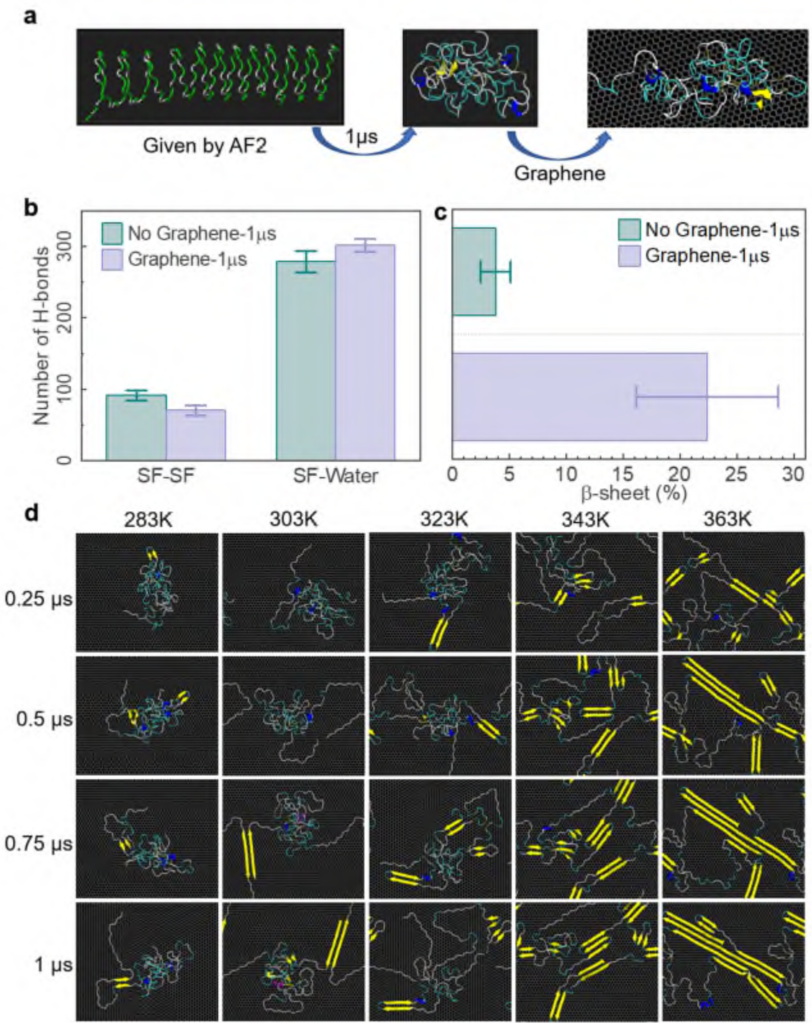

Supplementary Figure S18. The nucleation stage of SNF assembly on graphene. (a) The flow of all-atom MD simulation. (b) The average number with standard error of hydrogen bonds inside SF and between SF and water at 343 K after 1 μs assembly. (c) Simulated average β-sheet content with standard error of SF with or without graphene at 343 K after 1 μs assembly. It is important to underline that, within this 1 μs timeframe, conformational changes do not fully occur in either the systems with or without graphene. The purpose of comparing SF with and without graphene at 1 μs is to demonstrate that graphene can accelerate the assembly rate of SF. This finding suggests a templating effect of graphene in facilitating the conformational transition of SF. (d) Snapshots of SF assembly on graphene surface at different temperatures (283 K, 303 K, 323 K, 343 K, and 363 K) with different moments (0.25 μs, 0.5 μs,

0.75 μs and 1 μs). The data were collected from ten repeated simulations and expressed as as mean values +/- SEM.

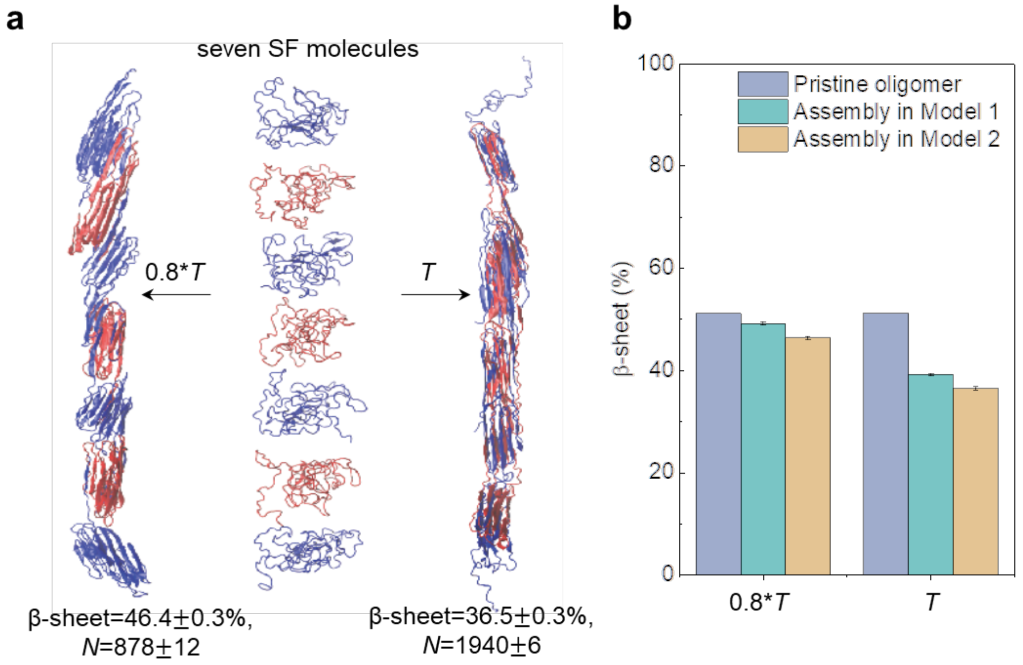

Supplementary Figure S19. Coarse-grained MD simulations. (a) Snapshots of model 2 at the growth stage. Two environment temperatures (i.e., 0.8**T* and *T*) are set. *N* is the average contact number. (b) Comparing average β-sheet content with standard error of assemblies, including graphene-induced oligomers at the 0-step stage and assemblies in model 1 and model 2 after 10^7 steps of assembling. The data were collected from five repeated simulations and expressed as as mean values +/- SEM.

Supplementary References

1. Yan, H. et al. Damping pathways of mid-infrared plasmons in graphene nanostructures. *Nat. Photon.* **7**, 394-399 (2013).
2. Avouris, T.L.a.P. Graphene Plasmonics for Terahertz to Mid-Infrared Applications. *ACS Nano* **8**, 1086-1101 (2014).
3. Semenenko, V. et al. Plasmon–Plasmon Interactions and Radiative Damping of Graphene Plasmons. *ACS Photonics* **5**, 3459-3465 (2018).
4. Hu, H. et al. Far-field nanoscale infrared spectroscopy of vibrational fingerprints of molecules with graphene plasmons. *Nat. Commun.* **7**, 12334 (2016).
5. García de Abajo, F.J. Graphene Plasmonics: Challenges and Opportunities. *ACS Photonics* **1**, 135-152 (2014).
6. Princen, J., Illingworth, J. & Kittler, J. A formal definition of the Hough transform: Properties and relationships. *J. Math. Imaging Vis.* **1**, 153-168 (1992).
7. Hu, X., Kaplan, D. & Cebe, P. Determining Beta-Sheet Crystallinity in Fibrous Proteins by Thermal Analysis and Infrared Spectroscopy. *Macromolecules* **39**, 6161-6170 (2006).
8. Lindahl, Abraham, Hess & Spoel, v.d., Vol. 2023, Edn. 2021.7 (Zenodo, 2023).
9. Miyamoto, S. & Kollman, P.A. Settle: An analytical version of the SHAKE and RATTLE algorithm for rigid water models. *J. Comput. Chem.* **13**, 952-962 (1992).
10. Hess, B., Bekker, H., Berendsen, H.J.C. & Fraaije, J.G.E.M. LINCS: A linear constraint solver for molecular simulations. *J. Comput. Chem.* **18**, 1463-1472 (1997).
11. Essmann, U. et al. A smooth particle mesh Ewald method. *J. Chem. Phys.* **103**, 8577-8593 (1995).
12. Bussi, G., Donadio, D. & Parrinello, M. Canonical sampling through velocity rescaling. *J. Chem. Phys.* **126**, 014101 (2007).
13. Berendsen, H.J.C., Postma, J.P.M., Gunsteren, W.F.v., DiNola, A. & Haak, J.R. Molecular dynamics with coupling to an external bath. *J. Chem. Phys.* **81**, 3684-3690 (1984).
14. Parrinello, M. & Rahman, A. Polymorphic transitions in single crystals: A new molecular dynamics method. *J. Appl. Phys.* **52**, 7182-7190 (1981).

15. Yang, S., Zhao, C., Yang, Y., Ren, J. & Ling, S. The Fractal Network Structure of Silk Fibroin Molecules and Its Effect on Spinning of Silkworm Silk. *ACS Nano* **17**, 7662-7673 (2023).
16. Ferrari, A.C. et al. Raman Spectrum of Graphene and Graphene Layers. *Phys. Rev. Lett.* **97**, 187401 (2006).
17. Wang, Y.y. et al. Raman Studies of Monolayer Graphene: The Substrate Effect. *J. Phys. Chem. C* **112**, 10637-10640 (2008).
18. Ni, Z., Wang, Y., Yu, T. & Shen, Z. Raman spectroscopy and imaging of graphene. *Nano Research* **1**, 273-291 (2008).
19. Suk, J.W. et al. Transfer of CVD-Grown Monolayer Graphene onto Arbitrary Substrates. *ACS Nano* **5**, 6916-6924 (2011).
20. Lin, Y.-C. et al. Clean Transfer of Graphene for Isolation and Suspension. *ACS Nano* **5**, 2362-2368 (2011).
21. Lin, Y.-C. et al. Graphene Annealing: How Clean Can It Be? *Nano Lett.* **12**, 414-419 (2012).
