# Supplementary material for: In-situ observation of silk nanofibril assembly via graphene plasmonic infrared sensor
Source: Nat Commun. 2024 May 31;15:4643. doi: 10.1038/s41467-024-49076-5 (PMC11143229; doi:10.1038/s41467-024-49076-5)
Supplement: Supplementary file 3 — Reporting Summary [file 41467_2024_49076_MOESM3_ESM.pdf]

nature portfolio

Corresponding author(s): Qing Dai, Xiaoxia Yang, Qian Wang, Shengjie Lin

Last updated by author(s): Apr 25, 2024

Reporting Summary

Nature Portfolio wishes to improve the reproducibility of the work that we publish. This form provides structure for consistency and transparency in reporting. For further information on Nature Portfolio policies, see our Editorial Policies and the Editorial Policy Checklist.

Statistics

For all statistical analyses, confirm that the following items are present in the figure legend, table legend, main text, or Methods section.

n/a	Confirmed	
☐	☒	The exact sample size (n) for each experimental group/condition, given as a discrete number and unit of measurement
☐	☒	A statement on whether measurements were taken from distinct samples or whether the same sample was measured repeatedly
☐	☒	The statistical test(s) used AND whether they are one- or two-sided<br>*Only common tests should be described solely by name; describe more complex techniques in the Methods section.*
☐	☒	A description of all covariates tested
☐	☒	A description of any assumptions or corrections, such as tests of normality and adjustment for multiple comparisons
☐	☒	A full description of the statistical parameters including central tendency (e.g. means) or other basic estimates (e.g. regression coefficient) AND variation (e.g. standard deviation) or associated estimates of uncertainty (e.g. confidence intervals)
☐	☒	For null hypothesis testing, the test statistic (e.g. F, t, r) with confidence intervals, effect sizes, degrees of freedom and P value noted<br>*Give P values as exact values whenever suitable.*
☐	☒	For Bayesian analysis, information on the choice of priors and Markov chain Monte Carlo settings
☐	☒	For hierarchical and complex designs, identification of the appropriate level for tests and full reporting of outcomes
☐	☒	Estimates of effect sizes (e.g. Cohen's d, Pearson's r), indicating how they were calculated

Our web collection on statistics for biologists contains articles on many of the points above.

Software and code

Policy information about availability of computer code

Data collection	The experimental data were collected by commercial instruments-atomic force microscope (Bruker Multimode 8) and infrared microscope (Nicolet iN10) with softwares (NanoScope Analysis and Omnic Picta). The simulation data were collected by free and open source software (PACKMOL, VMD 1.9.3, and OpenAWSEM). The deep learning algorithm code that support the findings of this study are available in the Source Data file.
Data analysis	The data analysis was done with the help of Origin (Version 2018pro) software.

For manuscripts utilizing custom algorithms or software that are central to the research but not yet described in published literature, software must be made available to editors and reviewers. We strongly encourage code deposition in a community repository (e.g. GitHub). See the Nature Portfolio guidelines for submitting code & software for further information.

Data

Policy information about availability of data

All manuscripts must include a data availability statement. This statement should provide the following information, where applicable:

- Accession codes, unique identifiers, or web links for publicly available datasets
- A description of any restrictions on data availability
- For clinical datasets or third party data, please ensure that the statement adheres to our policy

The data that support the findings of this study are provided in the Source Data file.

Research involving human participants, their data, or biological material

Policy information about studies with human participants or human data. See also policy information about sex, gender (identity/presentation), and sexual orientation and race, ethnicity and racism.

Reporting on sex and gender	/
Reporting on race, ethnicity, or other socially relevant groupings	/
Population characteristics	/
Recruitment	/
Ethics oversight	/

Note that full information on the approval of the study protocol must also be provided in the manuscript.

Field-specific reporting

Please select the one below that is the best fit for your research. If you are not sure, read the appropriate sections before making your selection.

[x] Life sciences [ ] Behavioural & social sciences [ ] Ecological, evolutionary & environmental sciences

For a reference copy of the document with all sections, see nature.com/documents/nr-reporting-summary-flat.pdf

Life sciences study design

All studies must disclose on these points even when the disclosure is negative.

Sample size	We used a sample size of 3-4 in experiment and 5-10 in simulation. We ensure that our sample size was sufficient to achieve statistically meaningful results.
Data exclusions	No data were excluded from the analyses conducted in our study. All collected data were included in the final analysis, and no exclusions were made.
Replication	We have conducted repeated molecular dynamic simulations, infrared spectroscopy and morphology measurements using various sensors and silk fibroin solutions to ensure the reproducibility of our experimental and simulation findings. All replication attempts were successful, confirming the reliability of our results.
Randomization	We utilized in-situ observation of silk nanofibril assembling on graphene nanoribbons to ensure that the allocation process was unbiased and controlled for any potential confounding factors.
Blinding	Blinding was not relevant in our study since we did in-situ observation of silk nanofibril assembling on graphene nanoribbons.

Reporting for specific materials, systems and methods

We require information from authors about some types of materials, experimental systems and methods used in many studies. Here, indicate whether each material, system or method listed is relevant to your study. If you are not sure if a list item applies to your research, read the appropriate section before selecting a response.

Materials & experimental systems

n/a	Involved in the study	
[x]	[ ]	Antibodies
[x]	[ ]	Eukaryotic cell lines
[x]	[ ]	Palaeontology and archaeology
[x]	[ ]	Animals and other organisms
[x]	[ ]	Clinical data
[x]	[ ]	Dual use research of concern
[x]	[ ]	Plants

Methods

n/a	Involved in the study	
[x]	[ ]	ChIP-seq
[x]	[ ]	Flow cytometry
[x]	[ ]	MRI-based neuroimaging

Plants

Seed stocks	/
Novel plant genotypes	/
Authentication	/
